# Gastrointestinal Perforation in Neonates: Aetiology and Risk Factors

**Published:** 2013-07-01

**Authors:** Ekwunife Okechukwu Hyginus, Ugwu Jideoffor, Modekwe Victor, Osuigwe Andrew N

**Affiliations:** Paediatric Surgery Unit, Nnamdi Azikiwe University Teaching Hospital Nnewi, Nigeria

**Keywords:** Neonate, Intestine, Perforation, Aetiology

## Abstract

Background: Gastrointestinal perforation (GIP) in neonates presents important challenges and mortality can be high. This is a report of recent experience with GIP in neonates in a developing country.

Patients and methods: A retrospective review of 16 neonates treated for GIP in a 3 year period.

Results: There were 9 males and 7 females, aged 0-28 days (median age =7days). Their weights at presentation ranged from 0.9 - 4.7kg (median =2.6). Five infants were premature. Twelve infants presented more than 72 hours after onset of symptoms. Plain abdominal radiographs showed peumoperitoneum in 9 infants. The cause of perforation was necrotising enterocolitis 6, intestinal obstruction 6, iatrogenic 3 and spontaneous 1. The site of perforation was ileum in 12 infants, stomach in 4 and colon in 4; 4 patients had involvement of more than one site. All the neonates underwent exploratory laparotomy with primary closure ( n=5) , resection and anastomosis( n=6), colostomy (n=3), Ileostomy ( n=2), partial gastrectomy (n=2) ,or gastrojejunostomy ( n=1). Two neonates had multiple procedures. Two very sick preterm babies had an initial peritoneal lavage. Surgical site infection is the commonest postoperative complication occurring in 9 infants. Anaesthesia sepsis and malnutrition is responsible for the seven deaths recorded.

Conclusions: Neonatal GIP has multiple aetiologies; NEC is the most common cause. Major mortality risk factors include NEC, multiple perforations, delayed presentation and prematurity.

## INTRODUCTION

Gastrointestinal perforation (GIP) in neonates presents important challenges and a high mortality of 15-70% has been reported [1-7]. Despite improvements in anaesthesia and neonatal intensive care, mortality has remained high, especially in the preterms [6]. In many developing nations, neonatal surgery is not yet well developed, and there are not many reports of GIP available from such regions. This is a report of the aetiology, risk factors and outcome from a referral hospital in Nigeria.

## MATERIALS AND METHODS

In little less than 4 years (January 2009 – November 2012), 16 neonates were treated at the Nnamdi Azikiwe University Teaching Hospital Nnewi, Nigeria. The hospital records of the infants have been retrospectively reviewed. 

## RESULTS

There were 9 males and 7 females, aged 0-28 days (median age= 7days). Their weights at presentation was 0.9 - 4.7kg (median=2.6kg). ) Five infants were premature. The interval between the onset of symptom and presentation was > 72hours in 12 infants (Table 1). Twelve neonates presented with varying levels of metabolic and cardiopulmonary derangement. One of them had associated gangrene of more than 75% of the anterior abdominal wall (Fig.1). The diagnosis of GIP was made based on clinical features, supported by plain X-ray (babygram) findings. All the infants were managed in the Special Care Baby Unit of the hospital. All received adequate resuscitation before surgery.

**Figure:**
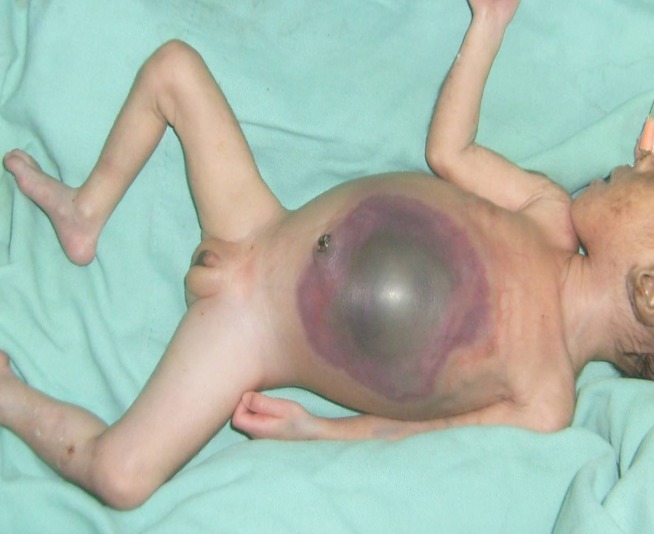
Figure 1:Abdominal wall necrosis

**Figure:**
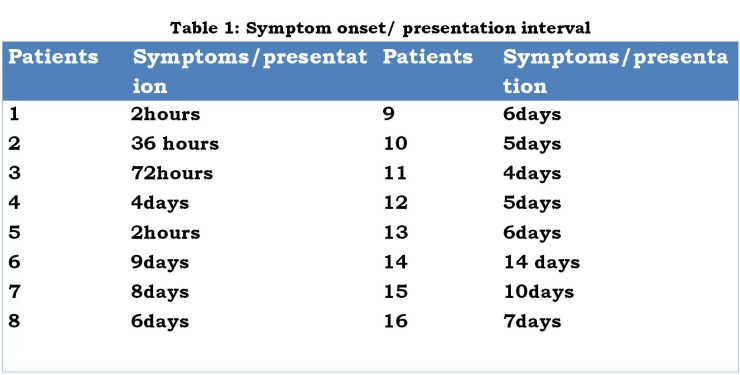
Table 1

Free intraperitoneal gas was noted on plain X-ray in 9 infants. 

At surgery, ileum was the commonest site of perforation (n=12). Others sites were stomach (n=4) and colon (n=4). Colonic perforations seen in babies with NEC were all associated with terminal ileal perforations. The only isolated colonic perforation was a caecal perforation in a baby with Ladd’s band tethering and occluding the ascending colon. 

In two babies with clinical evidence of perforation and pneumoperitoneum on X-ray, the perforation points were found sealed at laparotomy. Flag signs suggestive of perforation points with surrounding fibrinous exudates were found respectively in the ileum and stomach. 

Necrotizing enterocolitis was the cause of perforation in 6 infants: 4 of these infants were premature, 5 had multiple perforations, and all had been fed infant formula feeds prior to onset of symptoms. Obstructive pathologies causing perforation are shown in Table 2.

**Figure:**
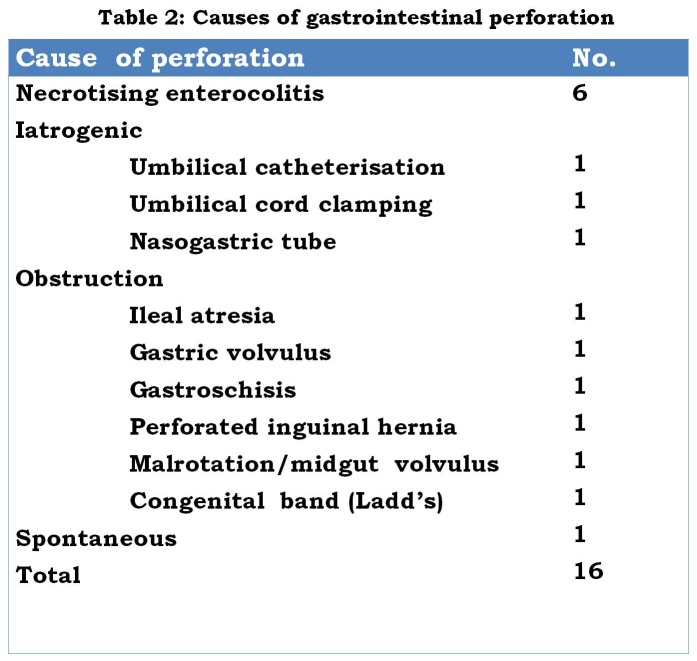
Table 2

Of the iatrogenic causes one child has a small intestinal perforation as well as injury to the right lobe of the liver caused by an umbilical catheter passed for intra venous fluid administration at the referring hospital. Another had perforation of the ileum from an umbilical cord clamp inadvertently applied by the delivery nurse over a hernia into the umbilical cord leading to gangrene. The third neonate had a perforated lower part of the greater curvature of the stomach from a nasogastric tube injury. 

The only child with spontaneous intestinal perforation (SIP) is a term neonate with a localized intestinal perforation (LIP) at the distal ileum. The single ileal perforation was about 1cm diameter located at the antimesenteric border. The gut wall surrounding the perforation appeared healthy. The baby was delivered of a diabetic mother by caesarean operation due to poor obstetric history. 

All the babies underwent exploratory laparotomy. Two very sick preterm babies had an initial peritoneal lavage and later delayed laparotomy due to non-improvement of their clinical state.

Corrective surgical procedures done include primary closure (n= 5) , resection and anastomosis(n=7), colostomy (n= 4), ileostomy (n=3), partial gastrectomy (n=2) and gastrojejunostomy (n=1). Two babies had more than one surgical procedure. Another two had repeat surgeries (enterostomies) for anastomotic failures.

Table 3 shows the postoperative complications encountered. In all, 7 babies died giving an overall mortality of 43.8%. Sepsis and malnutrition accounted for 3 deaths each while one death was anaesthesia related.

**Figure:**
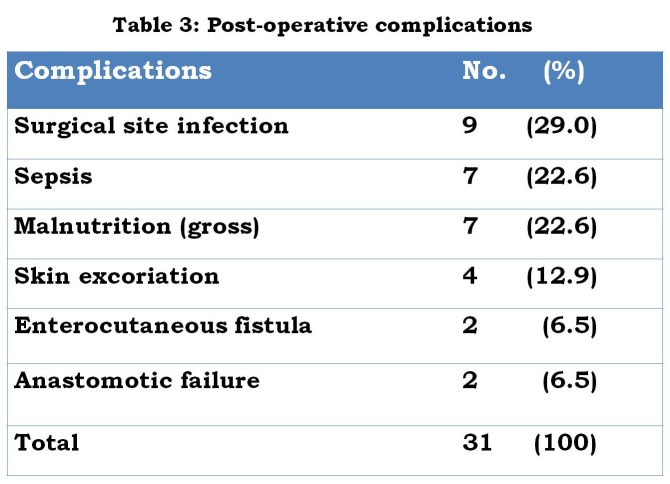
Table 3

## DISCUSSION

Necrotising enterocolitis (NEC) accounted for the major cause of neonatal GIP in our series. Worldwide, necrotizing enterocolitis (NEC) is the leading cause of neonatal GIP over other causes like mechanical obstruction and spontaneous intestinal perforation (SIP) [7]. As the survival of premature and critical ill neonates increase, the incidence of NEC is expectedly rising. Up to 90% of NEC occurs in preterms [8]. Intestinal perforation occurs in about 20% of those babies who develop NEC [9]. Perforation is often multiple. Exposure of neonates especially preterms with birth asphyxia to the high substrate load of artificial milk increases the risk of NEC and perforation [10]. In our series all the babies with perforation from NEC were found to have birth asphyxia and were also fed formula milk. 

Three of the perforations were from iatrogenic causes. A common factor in all these cases is the performance of the procedures by an inexperienced staff without proper supervision. Elhalaby et al. reported also a relatively high frequency of iatrogenic colorectal perforations (8.8%) [3]. In 2 of their cases, colonic perforation was caused by Nelton catheter inserted by an inexperienced nursing staff during colonic lavage. In addition, adverse perinatal conditions like asphyxia may create intestinal ischemia thus worsening the risk of perforation from iatrogenic injury [11]. All the neonates in our series with iatrogenic injury had birth asphyxia. 

A very worrisome fact is the late presentation of the neonates to the paediatric surgical unit. Majority of our cases present in a much compromised condition. This delay may be attributed to misdiagnosis and delay at the initial hospital of first contact. Morbidity and survival is poor for these late presenters.

Pneumoperitoneum in clinically suspicious patients is often taken as an indicator of bowel perforation and the need for a surgical intervention. However, pneumoperitoneum without perforation has been documented in babies that have been on mechanical ventilation, those with ruptured pneumatosis cystoides intestinalis, pneumomediastinum, or may be idiopathic [12]. Pneumoperitoneum is seen in about 63% of infants with GIP [13]. In our study, only 9 (56.3%) had intraperitoneal free gas. Absence of pneumoperitoneum on x-ray may be due to gas reabsorption. Also, naso-gastric decompression may also decompress the extra-luminal gas especially in wide perforations involving the stomach and proximal intestine. Apart from the large collection of gas beneath the diaphragm (cupola sign) diagnostic yield of X-ray for pneumoperitoneum can be improved by careful search for gas in other compartments: an oval gas shadow at the sub hepatic space obviously not in continuity with the rest of the bowel; a clear visualisation of the outer as well as the inner wall of a bowel loops (Rigler’s sign) and a small triangular collections of gas between loops of bowel (sign of triangle) [14, 15]. Also, absence or paucity of luminal gas shadows (gasless abdomen) may suggest perforation [16]. Absence of pneumoperitoneum does not however exclude perforation. Hence, good clinical suspicion is still very useful especially in resource poor setting like ours.

The possibility of spontaneous healing of gut perforations in neonates has been documented [11] and has led to some authors advocating initial conservative management for intestinal perforation [17]. In two of our patients with clinical evidence of perforation and pneumoperitoneum on x-ray, careful intraoperative search did not reveal any perforation points; instead only evidence of healed perforation was seen. Spontaneous healing of gut perforation is not very common. We currently explore all neonates with clinical and radiologic evidence of gut perforation as preoperative identification of infants with sealed perforation is not easy, and clinical features may be confusing. 

Peritoneal lavage is reserved for the very sick neonates. For those whose perforation was found to have healed spontaneously, post-operative survival was very good.

Mortality from neonatal intestinal perforation is still high, ranging from 40-70% [1, 3, 6, 11]. Some recent studies have however reported lower rates of between 30-36%[18, 19]. The persistent high mortality despite advancements in anaesthesia and neonatal intensive care has been attributed to increasing survival of extreme premature babies [5, 20]. Unlike NEC which accounts for a greater percentage of neonatal gastrointestinal perforations spontaneous intestinal perforation SIP) is rare and has a better prognosis. 

From our study NEC, prematurity, low birth weight, multiple perforations and delayed presentation are identifiable mortality risk factors of neonatal GIP (Table 4). Several other authors have documented similar findings [1, 3, 4, 6, 16]. 

**Figure:**
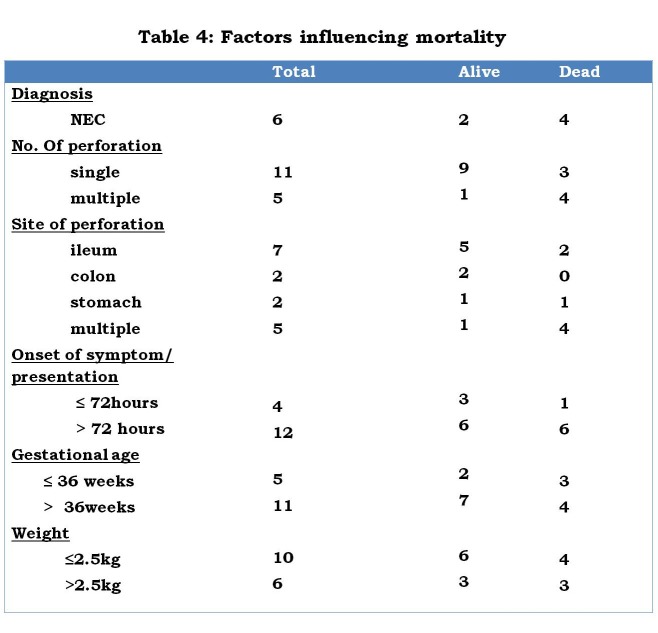
Table 4

Though not studied in this work, lack of state of the art neonatal intensive care unit facilities, dearth of specially trained personnel in neonatology and anaesthesia, absence of parenteral nutrition services are other critical factors that may be responsible for the high mortality. Many authors have documented these institutional factors to negatively affect neonatal surgical outcome in developing nations [21, 22]. Absence of these also contributed to the recorded high mortality rate in infants with multiple perforations who are especially at a higher risk of losing significant bowel lengths. It is also likely that the low number of very premature babies with perforation encountered in our study may be due to early demise at the referring centres due to lack of supportive facilities. 

In conclusion, neonatal GIP has multiple aetiologies; NEC is the most common cause. Major mortality risk factors include NEC, multiple perforations, delayed presentation and prematurity. Morbidity and mortality can be reduced by continued medical education of primary and secondary health care givers. This will improve the index of suspicion and thus early referral. Also, adequate supervision of nurses and junior doctors carrying out minor procedures and intubations will reduce the high number of iatrogenic cases. 

## Footnotes

**Source of Support:** Nil

**Conflict of Interest:** None

